# Multilevel determinants of physical violence among ever-partnered women in South Africa

**DOI:** 10.1007/s00737-024-01469-7

**Published:** 2024-05-11

**Authors:** Dikago Puoeng, Mluleki Tsawe

**Affiliations:** 1 Demography & Population Statistics Division, Statistics South Africa, Pretoria, South Africa; 2 Department of Population Studies and Demography, North-West University, Mahikeng Campus, South Africa; 3 Population and Health Research Focus Area, Faculty of Humanities, North-West University, Mahikeng Campus, South Africa

**Keywords:** Physical violence, Intimate partner violence, Ever-partnered women, Multilevel modelling, South Africa

## Abstract

**Background:**

Violence against women continues to be a challenge in many countries. Many women suffer physical violence at the hands of their intimate partners and sometimes this leads to their deaths. This study aimed to examine the multilevel determinants of physical violence among ever-partnered women in South Africa.

**Methods:**

We used data from the 2016 South Africa Demographic and Health Survey. The study has a weighted sample size of 4169 ever-partnered women aged 18–49 years, based on the domestic violence module. We included univariate, bivariate and multilevel logistic regression analysis. We included a two-level model to measure the relationship between the selected background characteristics and physical violence.

**Results:**

The prevalence of physical violence among ever-partnered women was 20.6%. The bivariate findings showed that educational status, employment status, witness to inter-parental violence, partner’s drinking habits, household wealth, educational difference, and province were statistically associated with physical violence. The multilevel analysis showed some evidence of between-cluster variation in physical violence. We found that age, education, employment status, witness to inter-parental violence, partner’s drinking habits, household wealth, education difference, place of residence, and province were key predictors of physical violence. The odds of physical violence were more than two-fold in the Eastern Cape and Mpumalanga compared to Gauteng.

**Conclusion:**

The study highlighted various key factors explaining physical violence. The findings suggest the need for targeted interventions aimed at specific communities of women, such as those from the Eastern Cape and Mpumalanga, as well as interventions that will empower women and address gender inequalities.

## Introduction

Globally, about one in three women have experienced either sexual or physical violence in their intimate relationships (World Health Organization 2013, 2021a). Although many countries have strategies in place for dealing with violence against women, intimate partner violence (IPV) continues to be a challenge in developing countries, particularly in sub-Saharan Africa (Ntoimo et al. 2021; Yaya et al. 2019). IPV is defined as actions by a current or former partner which results in psychological, physical, or sexual abuse; this includes behaviours such as psychological abuse, physical abuse, sexual coercion, and related behaviours (World Health Organization 2021b). Many countries tend to have different experiences of IPV. Recent studies in sub-Saharan Africa show differing percentages of IPV, with emotional violence at 29.40%, physical violence at 25.87%, and sexual violence at 18.75% (Chung-Ya et al. 2021; Jabbi et al. 2020; Muluneh et al. 2020). According to Pallitto et al. (2013), over 40% of women in Africa who had ever been in a relationship have ever experienced physical or sexual violence. Moreover, about 48% in Zambia have experienced violence from their partner, and 4–17% of women reported experiencing sexual violence (Kebede et al. 2022). In Ghana, 58% of women who have ever been married have experienced emotional violence, 40% have experienced physical violence, and 35% have experienced sexual violence (Tenkorang 2019). According to Bikinesi et al. (2017), more than one-third of women in Namibia have experienced physical or sexual violence. These statistics not only show that IPV continues to be a challenge in sub-Saharan Africa but there are differentials in the extent to which IPV is experienced between the countries.

Physical violence remains a serious social and human rights issue in South Africa. In some instances, physical violence leads to death. In recent times, intimate partner femicide has become a topical issue in the country with several women who have been killed by their intimate partners (Chadambuka and Warria 2019; Geldenhuys 2018; Mkhize and Sibisi 2022; Shai et al. 2022). The evidence shows that the prevalence of deaths due to IPV, particularly physical violence, continues to be high in the country, although there is evidence of a slight decrease between 1999 and 2017 (Abrahams et al. 2024; Mahaba 2022). In recent times, at least just before and around the Covid-19 period, newspaper reports on intimate partner femicide grew, suggesting an increase in violence against women as well as incidents of femicide (Matlhare 2021; Matshili 2018; Mniki 2020). Moreover, IPV-related deaths have been at the centre of various gender-based violence (GBV) campaigns in South Africa (Sibanda-Moyo et al. 2017). The South African government is part of various initiatives, such as the 16 days of activism these initiatives are aimed at reducing the prevalence of IPV in the country; the country also has a public holiday, Women’s Day, dedicated to women’s issues (Mbandlwa 2022; Peremore 2017). Moreover, there are various legislations which aim to combat IPV in the country, such as the Domestic Violence Act 116 of 1998 (South Africa 1999). The extent of intimate partner femicide calls for a better understanding of the factors determining physical violence in the country. Physical violence has far-reaching negative outcomes for the women who are abused, their families and the communities; besides leading to death, physical violence could also lead to injury, illness, and mental health issues (Habamenshi and Gasana 2022; Kumar et al. 2005; Oram et al. 2017).

Furthermore, there are various issues related to physical violence in the country. Traditional and cultural beliefs tend to influence behaviours related to physical violence, where men tend to believe that it is part of their culture to perpetrate such acts (Centre for the Study of Violence and Reconciliation 2016; Mesatywa 2014; Mshweshwe 2020). Cultural beliefs, inadequate community involvement in the fight against IPV, particularly violence against women, and political-religious powers tend to perpetuate patriarchy and male supremacy which maintains the status quo on IPV (Chadambuka and Warria 2019; Nadeem and Malik 2021). In many instances, women in some communities tend to be silent about IPV because they fear that speaking out will enrage the perpetrators and open room for more violence (Chadambuka and Warria 2019; Heron and Eisma 2021; Mesatywa 2014; Mshweshwe 2020). The cultural and social norms promote male dominance and act as barriers to reporting acts of IPV (Mshweshwe 2020). As such, women may not fully report the occurrences of physical violence, as well as the other forms of IPV, and this makes it difficult to comprehensively measure the prevalence of IPV (Palermo et al. 2014).

There are few studies focusing on examining the multilevel determinants of physical violence among women in South Africa. Various studies focusing on various forms of IPV and associated sociodemographic characteristics have been conducted in South Africa. Previous studies on various forms of IPV in the country have tended to focus on individual-level factors associated with IPV (Jewkes et al. 2002; Matseke et al. 2012, 2021; Mthembu et al. 2021; Pengpid and Peltzer 2014). Some of these studies have examined a mixture of different forms of IPV (e.g., emotional and sexual violence) (Mahlangu et al. 2022; Wood et al. 2008). Because physical violence occurs at individual and community levels, the multilevel modelling approach helps to increase our understanding of the interplay between the individual and community-level factors that are associated with physical violence. As a result, it is critical to view physical violence as a complex phenomenon involving interactions with both the individual and the community levels. Therefore, this study aimed to examine the determinants of physical violence among ever-partnered women in South Africa.

## Methods

### Data source

**Fig. 1:**
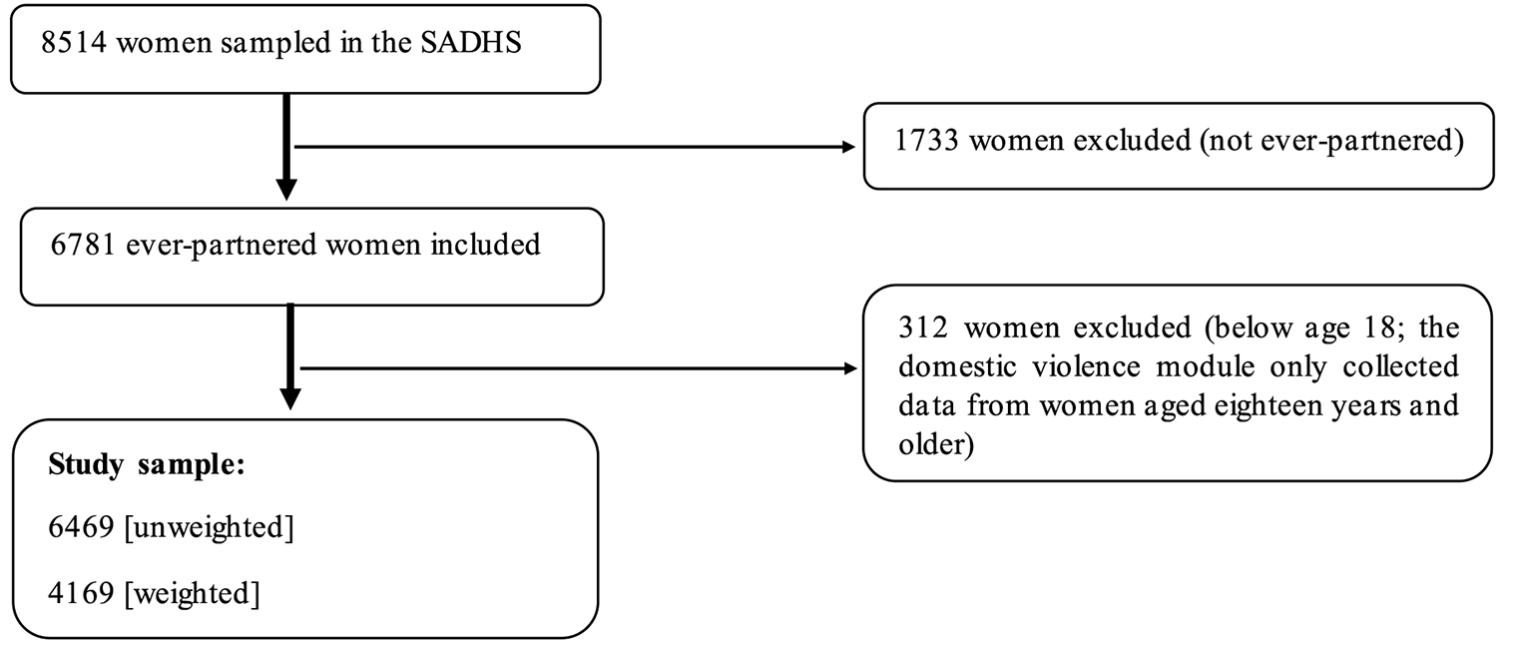
Schematic diagram for the selection of study participants

We used data from the South Africa Demographic and Health Survey (SADHS) conducted in 2016. This is the third standard DHS survey conducted in South Africa. The survey was conducted and coordinated by the National Department of Health in collaboration with Statistics South Africa, the South African Medical Research Council, and ICF (National Department of Health et al., 2019). The survey collected nationally representative data on various demographic and reproductive health indicators. The survey used probability proportional to size sampling, with a cross-sectional collection method, to collect information from households (National Department of Health et al., 2019). The SADHS questionnaire was pretested using classroom training and field practice; the field practice was done in selected provinces (National Department of Health et al., 2019). The response rate for the 2016 SADHS was 83%, with urban areas having lower response rates compared to non-urban areas (National Department of Health et al., 2019). We requested data from the DHSProgram and we were approved to download it for this paper. The data can be downloaded, upon registration and approval, from the DHSProgram website at https://tinyurl.com/24fn2fxb. The sample size for the study is based on a weighted sample of 4169 ever-partnered women who reported information about physical violence in the domestic violence module. Ever-partnered women are operationally defined as women who: (a) had a regular boyfriend/partner/fiancée, (b) were ever married or in-union (in-union meaning cohabiting), and (c) current have a boyfriend or had a boyfriend in the past. Further information about the selection of the study participants is on Fig. 1.

#### Outcome variable

We used ever-experienced physical violence as our outcome variable in the study. This variable was derived using five variables from questions asking women if (i) they have “ever been pushed, shook or had something thrown by husband/partner,” (ii) they have “ever been kicked or dragged by husband/partner,” (iii) they have “ever been strangled or burnt by husband/partner,” (iv) they have “ever been threatened with knife/gun or other weapon by husband/partner,” and (v) their “previous husband: ever hit, slap, kick or physically hurt respondent”. Women who responded with ‘often’, ‘sometimes’, ‘yes, but not in the last 12 months’, or ‘yes, but the frequency in last 12 months missing’ to any of the five variables were coded as 1 = Yes, or 0 = No otherwise. The outcome variable was created from variables labelled d105a, d105d, d105e, d105f, and d130a in the women’s file.

#### Explanatory variables

The explanatory variables selected for this study were age, educational status, employment status, witness to inter-parental violence, partner drinks alcohol, household wealth index, women’s decision-making autonomy, education difference, place of residence, and province. We briefly describe these variables in Table 1. These variables were included based on their previous statistical associations with physical violence in previous studies (Phiri et al. 2023; Skandro et al. 2024; Tiruye et al. 2020). These studies focused on various forms of IPV (while others focused on IPV as a whole, others focused on physical violence); for some of these studies, the unit of analysis was married women and not ever-partnered women. Also, none of these studies used the SADHS data.

**Table 1:** Description of the study variables

Explanatory variable	Description	Coding
*Individual-level factors*		
Age group	Age of respondent. These groups are based on variable v012 in the DSHS data.	1 = 18–19 2 = 20–24 3 = 25–29 4 = 30–39 5 = 40–49
Educational status	The highest level of education attained by the respondent. These groups are based on variable v106 in the DHS data.	0 = No education 1 = Primary 2 = Secondary+
Employment status	Current employment status of the respondent. This variable was derived from v717 on the DHS data.	0 = Unemployed 1 = Employed
Witness to inter-parental violence	Respondents were asked whether or not they had ever witnessed their father beat their mother. This variable is based on variable d121 in the DHS data.	0 = No 1 = Yes
Partners’ drinking habits	Respondents were asked whether or not their partners drank alcohol. This variable is based on variables d113 and d114 in the DHS data.	0 = Does not drink 1 = Drinks, never drunk 2 = Drinks, sometimes drunk 3 = Drinks, often drunk 8 = DNK
Household wealth	Household socioeconomic status. This variable is based on the wealth index, v190 in the DHS data. We combined the categories ‘poorest’ and ‘poorer’ into “poor” and also combined ‘richer’ and ‘richest’ into “rich”. More information about the variable can be obtained here: https://dhsprogram.com/topics/wealth-index/.	1 = Poor 2 = Average 3 = Rich
Women’s making-decision autonomy	This variable determines if women are involved in decisions about their health, purchases, and visits to family, either alone or jointly with their partner. It is based on v743a, v743b, and v743d in the DHS data.	0 = No 1 = Yes
Educational difference	Education difference between the respondents and their partners. This variable is based on a comparison between v715 and v133 in the DHS data.	1 = Partner is better educated 2 = Woman is better educated 3 = Equally educated 4 = Neither educated
*Community-level factors*		
Place of residence	Respondent’s place of residence. This is based on v025 in the DHS data.	1 = Urban 2 = Rural
Province	Respondent’s province of residence. This is based on v024 in the DHS data. Provinces such as Eastern Cape, KwaZulu-Natal, North West, Mpumalanga, and Limpopo are predominantly non-urban (i.e., rural and./or farm areas).	1 = Western Cape 2 = Eastern Cape 3 = Northern Cape 4 = Free State 5 = KwaZulu-Natal 6 = North West 7 = Gauteng 8 = Mpumalanga 9 = Limpopo

*Note* The DHSProgram has a guide for deriving these variables (on various statistical software programs) here: https://github.com/DHSProgram

### Statistical analysis

We used Stata version 14 for the analysis (StataCorp 2015). We conducted univariate, bivariate, and multivariate analyses. The study participants were first described using univariate analysis. A chi-square test (χ²) was used in bivariate analysis to assess the association between the explanatory variables and the outcome variable. The relationship between the multilevel components and physical violence was assessed using multivariate analysis (i.e., multilevel logistic regression). A two-level multilevel approach was applied in this study, with level one individuals nested within level two communities (the communities are based on 704 primary sampling units [clusters]). The analysis in model one looked at the relationship between individual-level factors and physical violence, while model two examines the relationship between community-level factors and physical violence among ever-partnered women. The probability of ever being physically harmed by an intimate partner can be estimated as follows:

$$\text{Log}\left[\frac{{\pi }_{ij}}{1-{{\pi }}_{ij}}\right]= {{\beta }}_{0}+{\beta }_{1}{X}_{ij}+{\beta }_{2}{Z}_{ij}+{\ddot{E}}_{j}+{e}_{ij}$$

where *i* represents the individual level and *j* represents the community level. The probability that the *i*^*th*^ woman in the *j*^*th*^ community may ever experience physical violence is represented by ${\pi }_{ij}$. The probability of not ever experiencing physical violence is represented by ${1-\pi }_{ij}$. The individual-level and community-level factors are denoted by X and Z, respectively. The intercept (${{\beta }}_{0}$) signifies the effect on the likelihood of ever experiencing physical violence when all explanatory variables in the model are set to zero or absent. The fixed coefficients are shown in terms of β’s. ${\pi }_{j}$denotes the effect of community-level factors on ever experiencing physical violence in the j^th^ community. The random errors occurring at the individual level are denoted by ${e}_{ij}$. Four models were fitted in this study. We used the null model, which excluded all explanatory factors, to examine the community-attributable variability of experiencing physical violence. Model 1 contained the individual-level factors. Model 2 contained the community-level factors. Model 3 contained both individual-level and community-level factors. Moreover, we used the intra-cluster correlation coefficient (ICC) using the linear threshold model (Merlo et al. 2006). A high ICC value indicates that the community clusters are relevant for the understanding of the individual experience of physical violence. There is a guiding document recommending how one can apply multilevel weights using the DHS data (Elkasabi et al. 2020). However, this recommendation does not work in the context of this study because the domestic violence module has a different sampling approach than the individual sampling used in the DHS; therefore, the multilevel analysis, and only this analysis, is not weighted. The formula for the ICC is denoted as follows:

$$ICC=\frac{{V}_{a}}{{V}_{a}+\frac{{\pi }^{2}}{3}}$$

Where ${V}_{a}$ is the variance between the primary sampling units in the null model and models 1-3, respectively. The $\frac{{\pi }^{2}}{3}$, which approximately equals to 3.29, is employed as the individual-level variance (Goldstein et al. 2002; Merlo et al. 2006). The median odds ratio (MOR), which is directly inversely proportional to the area-level variance, is the median value of the odds ratios between the areas with the highest and lowest risk when two areas are randomly selected (Tenny and Hoffman 2017). The MOR can be determined as follows:

$$MOR=(exp\sqrt{2\times {V}_{a }\times 0.6745 )} \approx \text{e}\text{x}\text{p}(0.95 \sqrt{{V}_{a }})$$

The proportional change in variance (PCV) looks at the change in the variance between the null model and the successive models (Merlo et al. 2006). It is expressed with the following formula:

$$PCV=\frac{{V}_{n-1}- {V}_{n-2}}{{V}_{n-1}}$$

Where ${V}_{n-1}$ is the PSU variance of the null model and ${V}_{n-2}$ is the PSU variance of subsequent models (1, 2, and 3, respectively). When the PCV value is less than 100%, it indicates that, compared to the null model, adding individual-level factors into the model does not fully replace the PSU variance. For instance, if the PCV value is 70%, this means that approximately 30% of the observed variation in physical violence at the individual level can be attributed to differences between the PSUs. To evaluate the overall fit between the model and the data, we employed the deviance (-2LL) and the Akaike Information Criterion (AIC). We checked for collinearity using the variance inflation factor (VIF); the mean VIF was 1.23, with a minimum VIF of 1.02 and a maximum VIF of 1.49.

## Results

### Socio-demographic characteristics

The descriptive analysis of the study participants, based on a weighted sample of 4169 ever-partnered women, is presented in Table 2. The results showed that the majority (32.0%) of participants were between the ages of 30–39, followed by those between the ages of 40–49 (23.1%). In terms of education, 2.1% of respondents had no education, 8.7% had only a primary education, and 89.1% had a secondary education. Only 41.1% of the women were employed, while 58.9% were not employed. Additionally, 14.5% of the women reported having witnessed inter-parental violence, whereas 85.5% of the women said they had not witnessed inter-parental violence. Regarding the partners’ drinking habits, the majority of the women (56.4%) reported that their partners did not drink. There was also a large percentage of women (34.9%) who reported that their partners drank alcohol and were sometimes drunk. The majority of the women (39.9%) were from poor households, followed by those from rich households (38.9%), and the least were from average-wealth households (21.3%). Moreover, the majority (61.9%) of women reported that they did not have decision-making autonomy. Furthermore, in terms of educational differences between themselves and their partners, the majority of women (71.1%) reported that their partner was better educated. In terms of geographical location, there were more women (67.9%) from urban areas. Likewise, the majority (28.3%) of women were from the Gauteng province.

**Table 2:** Distribution of respondents and prevalence of physical violence use by explanatory factors

Variable	Experienced physical violence				*N* (%)	χ^2^	
	No		Yes			value	*p*-value
	%	CI	%	CI			
*Individual-level factors*							
**Age**						9.0	0.062
18–19	81.5	[72.8–87.9]	18.5	[12.1–27.2]	236 (5.7)		
20–24	82.7	[79.0-85.8]	17.3	[14.2–21.0]	805 (19.3)		
25–29	78.4	[74.2–82.1]	21.6	[17.9–25.8]	831 (19.9)		
30–39	77.8	[74.5–80.7]	22.2	[19.3–25.5]	1336 (32.0)		
40–49	79.2	[75.4–82.7]	20.8	[17.3–24.6]	962 (23.1)		
**Educational status**						22.2	0.000
No education	80.4	[69.4–88.1]	19.6	[11.9–30.6]	89 (2.1)		
Primary	69.2	[62.7–75.1]	30.8	[24.9–37.3]	364 (8.7)		
Secondary+	80.4	[78.2–82.4]	19.6	[17.6–21.8]	3716 (89.1)		
**Employment status**						5.7	0.017
Not employed	80.4	[78.2–82.5]	19.6	[17.5–21.8]	2457 (58.9)		
Employed	77.9	[74.4–81.0]	22.1	[19.0-25.6]	1712 (41.1)		
**Witness to inter-parental violence**						149.8	0.000
No	82.9	[81.0-84.6]	17.1	[15.4–19.0]	3566 (85.5)		
Yes	58.8	[52.7–64.6]	41.2	[35.4–47.3]	603 (14.5)		
**Partner’s drinking habits**						232.7	0.000
Does not drink	86.3	[84.1–88.2]	13.7	[11.8–15.9]	2350 (56.4)		
Drinks, never drunk	82.7	[64.0-92.8]	17.3	[7.2–36.0]	44 (1.1)		
Drinks, sometimes drunk	74.3	[71.0-77.4]	25.7	[22.6–29.0]	1454 (34.9)		
Drinks, often drunk	50.2	[42.2–58.2]	49.8	[41.8–57.8]	311 (7.5)		
DNK	91.8	[68.4–98.3]	8.2	[1.7–31.6]	10 (0.2)		
**Household wealth**						23.0	0.000
Poor	76.2	[73.2–79.0]	23.8	[21.0-26.8]	1662 (39.9)		
Average	79.5	[75.5–83.1]	20.5	[16.9–24.5]	887 (21.3)		
Rich	82.6	[79.5–85.3]	17.4	[14.7–20.5]	1620 (38.9)		
**Decision-making autonomy**						1.4	0.236
No	78.7	[76.2–81.0]	21.3	[19.0-23.8]	2580 (61.9)		
Yes	80.5	[77.8–82.9]	19.5	[17.1–22.2]	1589 (38.1)		
**Education difference**						11.5	0.009
Partner is better educated	79.1	[76.8–81.2]	20.9	[18.8–23.2]	2963 (71.1)		
Woman is better educated	76.2	[71.5–80.4]	23.8	[19.6–28.5]	615 (14.8)		
Equally educated	84.6	[79.7–88.5]	15.4	[11.5–20.3]	552 (13.2)		
Neither educated	82.8	[63.4–93.1]	17.2	[6.9–36.6]	39 (0.9)		
*Community level factors*							
**Place of residence**						0.3	0.558
Urban	79.7	[77.1–82.0]	20.3	[18.0-22.9]	2831 (67.9)		
Rural	78.9	[75.4–81.9]	21.1	[18.1–24.6]	1338 (32.1)		
**Province**						105.3	0.000
Western Cape	75.6	[69.4–80.9]	24.4	[19.1–30.6]	452 (10.8)		
Eastern Cape	66.3	[61.2–71.0]	33.7	[29.0-38.8]	450 (10.8)		
Northern Cape	78.8	[73.2–83.5]	21.2	[16.5–26.8]	83 (2.0)		
Free State	78.0	[72.8–82.5]	22.0	[17.5–27.2]	223 (5.3)		
KwaZulu-Natal	84.8	[80.8–88.1]	15.2	[11.9–19.2]	750 (18.0)		
North West	70.5	[61.6–78.1]	29.5	[21.9–38.4]	297 (7.1)		
Gauteng	85.0	[80.0–89.0]	15.0	[11.0–20.0]	1179 (28.3)		
Mpumalanga	72.8	[68.2–77.0]	27.2	[23.0-31.8]	350 (8.4)		
Limpopo	85.2	[80.8–88.7]	14.8	[11.3–19.2]	385 (9.2)		
**Total**	**79.4**	**[77.4–81.3]**	**20.6**	**[18.7–22.6]**	**4169 (100.0)**		

*Note* CI = confidence interval; DNK = do not know

### Prevalence of ever experiencing physical violence

Table 2 shows the prevalence of physical violence among women by explanatory factors. The findings showed that educational status, employment status, witness to inter-parental violence, partner’s drinking habits, household wealth, educational difference, and province were associated with physical violence. Women aged 30–39 had a higher prevalence (22.2%) of physical violence, followed by those aged 25–29 (21.6%). Physical violence was less common among women under the age of 25. The prevalence of physical violence was higher (30.8%) among women with primary education. Women who were employed had a higher prevalence (22.1%) of physical violence, while it was lower (19.6%) among women who were not employed. Moreover, the prevalence of physical violence was higher (41.2%) among women who witnessed inter-parental violence. The level of physical violence increased with the partner’s level of drunkenness. Women whose partners drank alcohol and were often drunk had a higher prevalence (49.8%) of physical violence. The experience of physical violence decreased with increasing household wealth; women from poor households had a higher prevalence (23.8%) of physical violence, while it was lower (17.4%) among women from rich households.

Additionally, women who had no decision-making autonomy had a higher prevalence (21.3%) of physical violence. Surprisingly, women who were better educated than their partners had a higher prevalence (23.8%) of physical violence; likewise, women whose partners were better educated had a higher prevalence (20.9%) of physical violence. However, the prevalence of physical violence was lower (15.4%) among women who had the same educational level as their partner. In terms of geographical location, women from rural areas had a slightly higher prevalence (21.1%) of physical violence, while it was a bit lower (20.3%) among women from urban areas. Generally, this rural-urban difference in physical violence against women is miniscule. In terms of the province, women from Eastern Cape (33.7%), North West (29.5%), and Mpumalanga (27.2%) had a higher prevalence of physical violence, while it was lower among women from Limpopo (14.8%), Gauteng (15.0%), and KwaZulu-Natal (15.2%).

### Determinants of physical violence

Table 3 presents the fixed and random effects results for physical violence among ever-partnered women in South Africa. The null model showed some variation in physical violence across the clusters (ICC = 11.0%; variance = 0.407 [95% CI = 0.27–0.62]). The variation (ICC) decreased between the models, to 7.1% in model one, then 6.7% in model two, and 4.6% in model three. In the full model, the MOR of 1.5 is low because it relates to an ICC of 0.046 (4.6%). This means that only 4.6% of the variation in physical violence is due to between-cluster differences. Also, in model three, the PCV suggested that the individual and community-level factors accounted for about 60.9% of the variation observed in physical violence among ever-partnered women in South Africa. The deviance as well as the AIC was lowest for model three, suggesting that this was the best-fitted model. We interpret the fixed effects results of the best-fitted model below. The fixed effects results showed that women in their late 20s and those in their 30s had higher odds of physical violence in South Africa. Women aged 25–29 [AOR: 1.30, 95% CI: 1.01–1.68] as well as those aged 30–39 [AOR: 1.30, 95% CI: 1.05–1.61] had higher odds of physical violence compared to women aged 40–49. Women with secondary or higher education had lower odds [AOR: 0.72, 95% CI: 0.56–0.94] of physical violence compared to those with primary education. Moreover, women who were employed had higher odds [AOR: 1.21, 95% CI: 1.02–1.44] of physical violence compared to those who were not employed. Likewise, women who had witnessed inter-parental violence when they were younger had higher odds [AOR: 2.74, 95% CI: 2.24–3.37] of physical violence compared to those who had not witnessed inter-parental violence.

**Table 3:** Multilevel determinants of physical violence among ever-partnered women in South Africa

Characteristics	Model 0	Model 1	Model 2	Model 3
	AOR [95% CI]	AOR [95% CI]	AOR [95% CI]	AOR [95% CI]
*Individual-level factors*				
**Age**				
18–19		1.12 [0.71–1.77]		1.09 [0.69–1.73]
20–24		1.00 [0.76–1.33]		1.00 [0.75–1.32]
25–29		1.31 [1.01–1.69]		1.30* [1.01–1.68]
30–39		1.28 [1.03–1.60]		1.30* [1.05–1.61]
40–49®		1		1
**Educational status**				
No education		0.61 [0.33–1.12]		0.61 [0.33–1.12]
Primary®		1		1
Secondary+		0.70 [0.54–0.91]		0.72* [0.56–0.94]
**Employment status**				
Not employed®		1		1
Employed		1.21 [1.02–1.44]		1.21* [1.02–1.44]
**Witness to inter-parental violence**				
No®		1		1
Yes		2.93 [2.38–3.59]		2.74*** [2.24–3.37]
**Partner’s drinking habits**				
Does not drink		0.49 [0.41–0.59]		0.52*** [0.44–0.62]
Drinks, never drunk		0.79 [0.33–1.89]		0.72 [0.30–1.73]
Drinks, sometimes drunk®		1		1
Drinks, often drunk		2.63 [2.01–3.45]		2.60*** [1.99–3.39]
DNK		0.47 [0.09–2.40]		0.50 [0.10–2.57]
**Household wealth**				
Poor®		1		1
Average		0.81 [0.66-1.00]		0.75** [0.60–0.93]
Rich		0.63 [0.51–0.78]		0.55*** [0.43–0.69]
**Decision-making autonomy**				
No®		1		1
Yes		1.04 [0.84–1.29]		1.03 [0.84–1.28]
**Education difference**				
Partner is better educated		0.92 [0.71–1.18]		0.93 [0.72–1.20]
Woman is better educated®		1		1
Equally educated		0.68 [0.50–0.92]		0.68* [0.50–0.93]
Neither educated		0.70 [0.25–1.97]		0.69 [0.24–1.94]
*Community level factors*				
**Place of residence**				
Urban			1.08 [0.89–1.33]	1.42* [1.14–1.78]
Rural®			1	1
**Province**				
Western Cape			1.78 [1.17–2.70]	1.88* [1.23–2.87]
Eastern Cape			3.35 [2.29–4.90]	2.71*** [1.84–3.98]
Northern Cape			1.60 [1.05–2.42]	1.51 [0.99–2.31]
Free State			1.89 [1.27–2.82]	1.86* [1.25–2.79]
KwaZulu-Natal			1.19 [0.80–1.78]	1.27 [0.85–1.91]
North West			2.18 [1.46–3.26]	1.79*** [1.19–2.69]
Gauteng®			1	1
Mpumalanga			2.73 [1.86–4.02]	2.54*** [1.72–3.74]
Limpopo			1.10 [0.72–1.67]	1.32 [0.86–2.03]
**Random effects result**				
PSU variance (95% CI)	0.407 [0.27–0.62]	0.252 [0.14–0.46]	0.235 [0.13–0.43]	0.159 [0.07–0.37]
ICC %	11.0	7.1	6.7	4.6
MOR	1.8	1.6	1.6	1.5
PCV %	®	38.1	42.3	60.9
**Model fitness**				
-2LL	4212	3860	4133	3803
AIC	4216	3900	4155	3861
PSU	704	704	704	704

*Note* * = *p* < 0.05; ** = *p* < 0.01; *** = *p* < 0.001; ® = reference category; AOR = adjusted odds ratio; CI = confidence interval; DNK = do not know; ICC = intra-cluster correlation coefficient; MOR = median odds ratio; PCV = proportional change in variance; -2LL = deviance [-2 log-likelihood]; AIC = Akaike Information Criterion; PSU = Primary Sampling Unit

The findings further showed that a partner’s alcohol consumption plays a significant role in women’s experience of physical violence. Women whose partners did not drink alcohol had lower odds [AOR: 2.74, 95% CI: 2.24–3.37] of physical violence compared to those whose partners drank alcohol and were sometimes drunk. Additionally, women whose partners drank alcohol and were often drunk had higher odds [AOR: 2.60, 95% CI: 1.99–3.39] of physical violence compared to those whose partners drank alcohol and were sometimes drunk. Moreover, women’s experience of physical violence decreased with increasing household wealth status. Women from average-wealth households had lower odds [AOR: 0.75, 95% CI: 0.60–0.93] of physical violence compared to those who were from poor households. Likewise, women from rich households had lower odds [AOR: 0.55, 95% CI: 0.43–0.69] of physical violence compared to those who were from poor households. In terms of the educational difference between the woman and partner, women who were equally educated with their partner had lower odds [AOR: 0.68, 95% CI: 0.50–0.93] of physical violence compared to those who were better educated than their partner. In addition, women’s geographical location played an important role in their odds of ever experiencing physical violence from an intimate partner. Women from urban areas had higher odds [AOR: 1.42, 95% CI: 1.14–1.78] of physical violence compared to those from rural areas. Furthermore, women from Eastern Cape [AOR: 2.71, 95% CI: 1.84–3.98], Mpumalanga [AOR: 2.54, 95% CI: 1.72–3.74], Western Cape [AOR: 1.88, 95% CI: 1.23–2.87], Free State [AOR: 1.86, 95% CI: 1.25–2.79], and North West [AOR: 1.79, 95% CI: 1.19–2.69] had higher odds of physical violence compared to those from Gauteng.

## Discussion

This study aimed to examine the factors determining physical violence among ever-partnered women in South Africa. We found some between-cluster variation in physical violence in the country. The findings revealed that level of education, employment status, witness to inter-parental violence, partners’ drinking habits, household wealth, education difference, and province, were associated with physical violence among ever-partnered women. IPV has been linked to these factors in several studies, focusing on various sub-Saharan African countries (Ahinkorah et al. 2018; Jabbi et al. 2020; Kebede et al. 2022; Tenkorang 2019; Tiruye et al. 2020). We found that women in their late twenties and those in their thirties had higher odds of experiencing physical abuse. This finding is in line with studies conducted in other developing countries that found women aged thirty-five years and older were more likely to experience violence at the hands of a spouse (Chernet and Cherie 2020; Lacey et al. 2016). However, other studies (Ahinkorah et al. 2018; Issahaku 2017; Warren 2015) have discovered contradictory findings, specifically that younger women were more likely to experience greater physical abuse than older women. Our finding could be explained by that in South Africa, women are usually in intimate relationships with partners who are significantly older than them (Jeawon 2023; Shefer and Strebel 2012), where the power dynamics could make the controlling behaviour and abuse more prevalent.

Moreover, we found that, in contrast to women with primary education, those with secondary education or higher had lower odds of experiencing physical violence. The findings are similar to those of a study by Ahinkorah et al. (2018), which found that education empowers women to have a better understanding of their rights and the rights of other women in their communities, which has the potential to reduce physical violence. The prevalence of physical violence is usually higher among women with lower levels of education, (Kapiga et al. 2017). Moreover, women with lower levels of education tend to have lower socio-economic status, which may explain their increased likelihood of experiencing physical violence. Women in the sub-Saharan African region have low literacy levels, which increases their exposure to the patriarchal male control that permeates their societies and increases their risk of experiencing physical violence (Muluneh et al. 2021). Furthermore, education is an important empowerment factor that has the potential to reduce the perpetrators’ power over women and thus increase their autonomy. Being educated also reduces women’s dependence on their perpetrators and limits their chances of being victims of physical violence (Conroy 2014).

Surprisingly, we found that employed women had higher odds of physical violence compared to those who were unemployed. This is an interesting finding when looked at in conjunction with one for education above; while better education seems to decrease physical violence being employed seems to increase it. Although we do not have a definitive answer for this phenomenon, there is a possibility that many factors could be at play here (i.e., traditional roles where men are providers and may be in control of most of the decisions in the households). Also, we are not able to tell whether those who are employed are also educated. Moreover, one would think that employment promotes autonomy and allows women to leave abusive situations. However, employed women are more independent and less inclined to adhere to social norms that dictate that women should be subservient to their spouses (Jabbi et al. 2020); in a patriarchal society, this ‘autonomy’ changes the traditional gender power dynamics in the family, making such women’s relationships violent because their partners feel the need to have some control in those relationships (Jabbi et al. 2020). Likewise, Khan and Klasen (2018), argue that in a society where men dominate, employed women are likely to be educated and tend to reject traditional gender roles, which could be viewed as a transgression, thus leading to IPV. Moreover, our findings are in line with other studies from sub-Saharan Africa, which found that being employed is associated with physical violence among women in this region (Ahinkorah et al. 2018; Alangea et al. 2018; Gage and Thomas 2017). However, other studies have found that unemployed women are more likely to conceal their violent experiences due to worries about family strife, and also tend to be reliant on their partners for financial support (Biswas 2017; Tenkorang 2018).

We also found that witnessing inter-parental violence as a child is a significant factor in experiencing physical violence later in life. Women who had witnessed inter-parental violence had higher odds of physical violence. Witnessing parental abuse as a child can have long-term effects on interpersonal interaction problems (Afe et al. 2016; Tu and Lou 2017). Being exposed to parental violence from a young age has the potential to cause long-lasting trauma (Kieselbach et al. 2021), and can increase the odds of a woman experiencing violence from their partner in adulthood (Aboagye et al. 2022). Moreover, certain behaviours, such as alcohol consumption by the woman or their partner (or both) have negative effects and can lead to physical violence in cases where there is alcohol abuse. We found that women whose partners drank alcohol and were sometimes drunk had higher odds of physical violence. Various studies have found that women who have partners who abuse alcohol are more likely to report physical violence (Adebowale 2018; Greene et al. 2017; Mthembu et al. 2021; Tanimu et al. 2016). According to Parrott and Eckhardt (2018), alcohol is known to have cognitive and behavioural effects, such as behavioural disinhibition, which worsens aggression.

Additionally, we found that physical violence decreases with household wealth; women with a lower household wealth status had higher odds of physical violence. This may be explained by the fact that wealthy women are more independent in their decision-making, but poorer women are more likely to experience physical violence because of their dependence on a partner to provide for their basic livelihood (Angaw et al. 2021). We also found that women who were as equally educated as their partners had lower odds of physical violence. This is in line with a study by (Tiruye et al. 2020) which also found lower odds of IPV among women who had the same education as that of their partners. Being equally educated can promote some level of ‘equality’ in the relationship, thereby decreasing the chances of physical violence. Furthermore, we found that geographical location plays an important role in the experience of physical violence. Women from urban areas had higher odds of physical violence. A study by (Nakitto et al. 2023) also found similar results where women from rural areas had reduced odds of experiencing IPV. In South Africa, the majority of the population resides in urban areas, which could explain this finding (Statistics South Africa 2023). Furthermore, we found that women from the Western Cape, Eastern Cape, Free State, North West, and Mpumalanga had higher odds of physical violence compared to those from Gauteng. Specifically, physical violence was highest in Eastern Cape and Mpumalanga. Other studies also show that there are often notable regional variations in the likelihood of experiencing IPV in various countries (Mshweshwe 2020; Ross et al. 2021). This finding, particularly in the case of the Eastern Cape and Mpumalanga, may be explained by the fact that these two provinces are mostly rural (non-urban) provinces where highly patriarchal practices, such as wife/partner-beating, are still practised (Mills 2005).

### Strengths and limitations of the study

This study has some strengths and limitations. Due to the sensitive nature of physical violence, the respondents may be reluctant to share their genuine experiences of physical violence; as a result, there may be some social desirability bias or reporting bias in the reported findings. Moreover, the cross-sectional nature of this research restricts the application of causal inferences (for example, one cannot assert that alcohol intake causes physical violence). Furthermore, this study has several strengths. Among these is that the way in which the questions are asked in the DHS allows women to describe the abusive behaviours they have experienced, instead of asking direct questions and having words such as ‘abuse’ or ‘violence’ in the questions; direct questions on this sensitive topic can lead to under-reporting as women may not want to acknowledge their experience of violence when such words are used in the questions. Because we only focused on physical violence, the findings of this study should not be interpreted as encompassing all the forms of IPV. However, this study contributes to the literature by examining the multilevel determinants of physical violence. Looking at this important topic beyond the individual level is important in understanding its complexity in the African context. Also, we use multilevel modelling which allows for better statistical inference about the communities (PSUs) from which the women come.

## Conclusion

The findings of this study have highlighted several important factors influencing physical violence among ever-partnered women in South Africa, with significant public health and policy implications. Although South Africa has various policies and legislations advocating for the elimination of violence against women, it seems that those policies are either not adequate or are not properly implemented because physical violence against women continues to be a problem. This study found various key determinants of physical violence in South Africa. The findings suggest that physical violence against women is multifaceted and needs context-specific interventions at the societal level that are aimed at addressing gender inequality and promoting women’s empowerment. Therefore, we recommend that the policies and programmes aimed at reducing physical violence focus on underlying factors which promote gender inequality, such as poverty as well as lack of education; there is also a need for policies that focus on men’s behaviour towards women and their alcohol abuse to reduce physical violence. Moreover, policies aimed at promoting education and employment, particularly for women in their twenties and thirties, may help alleviate the risk of experiencing abuse. Furthermore, there is a need for in-depth qualitative studies which will focus on understanding the possible cultural norms promoting physical violence in predominantly rural provinces, with the highest odds of physical violence, such as the Eastern Cape and Mpumalanga province. Moreover, such future studies could also look at the nuanced power dynamics and control within relationships in trying to understand the reasons for employed women having higher odds of physical violence compared to those who were unemployed.
